# Genetic determinants of growth hormone and GH-related phenotypes

**DOI:** 10.1186/s12864-017-4219-z

**Published:** 2017-10-24

**Authors:** Erik Hallengren, Peter Almgren, Malin Svensson, Widet Gallo, Gunnar Engström, Margaretha Persson, Olle Melander

**Affiliations:** 1 From the Department of Clinical Sciences, Lund University, Malmö, Sweden; 2 Department of Internal Medicine, Skåne University Hospital, 205 02 Malmö, SE Sweden

**Keywords:** Growth hormone, GWAS, Height, SNP, Coronary artery disease

## Abstract

**Background:**

Higher fasting Growth Hormone (GH) has been associated with increased cardiovascular morbidity and mortality. Our objective was to find genetic determinants of fasting GH in order to facilitate future efforts of analyzing the association between fasting growth hormone and cardiovascular disease. A genome-wide association study (GWAS) was performed in a discovery cohort of 4134 persons (58% females; age 46–68 yrs), linking SNPs to fasting hs-GH. Fifteen SNPs were replicated in an independent cohort of 5262 persons (28.9% females; age 56–85 yrs). The best performing SNP was analyzed vs GH-related variables in a third independent cohort (*n* = 24,047; 61% females; age 44–73 yrs). A candidate gene approach searched for significant SNPs in the genes GH1 and GHR in the discovery cohort and was replicated as previously described.

**Results:**

In the GWAS, the minor allele of rs7208736 was associated with lower GH in the discovery cohort (*p* = 5.15*10^-6) and the replication cohort (*p* = 0.005). The GH reducing allele was associated with lower BMI (*P* = 0.026) and waist (*P* = 0.021) in males only. In the candidate gene approach rs13153388 in the GHR-gene was associated with elevated GH-levels (*P* = 0.003) in the discovery cohort only and reduced height (*P* = 0.003).

**Conclusion:**

In the first GWAS ever for GH, we identify a novel locus on chromosome 17 associated with fasting GH levels, suggesting novel biological mechanisms behind GH secretion and GH-related traits. The candidate gene approach identified a genetic variant in the GHR, which was associated with an elevation of fasting hs-GH and lower height suggesting reduced GHR ligand sensitivity. Our findings need further replication.

**Electronic supplementary material:**

The online version of this article (10.1186/s12864-017-4219-z) contains supplementary material, which is available to authorized users.

## Background

Growth hormone (GH) is the main regulator of longitudinal growth during childhood and adolescence. In addition, this versatile hormone has effects on lipolysis in adipose tissue, protein anabolism in muscle cells and glucose and lipid metabolism throughout the entire life [1]. We recently showed that high fasting levels of growth hormone are associated with cardiovascular disease and that higher levels predict cardiovascular morbidity and mortality independently of traditional cardiovascular risk factors confirming previous reports from the 1990’s [2, 3]. However, whether or not this association is causal is unknown. Causality can be assessed by randomized controlled trials (RCTs), administering GH or blocking its effect, and test if such treatment affects the risk of cardiovascular disease. However, such studies are unlikely to take place due to large costs. In light of this, Mendelian Randomization studies have established themselves as a cost effective alternative to RCTs as they have convincingly been shown to be able to assess causality between circulating biomarkers and medical traits and diseases [4, 5]. In order to enable tests of causality between GH and cardiometabolic diseases using Mendelian Randomization, we here set out to identify common genetic variants contributing to fasting GH concentration.

GH is secreted in pulses, which makes measurement difficult. However, the levels of GH are quite low and stable during the morning hours [2, 6–8], which enable measurement of fasting GH concentrations. Even if the variability in single values of fasting GH is large, there is a strong positive association to 24-h GH production on a group level [9].

The aim of this study was to find genetic determinants of the fasting level of growth hormone. We applied two different strategies, the first being a GWAS-approach where we related single nucleotide polymorphisms (SNPs) from the whole genome to hs-GH in the Cardiovascular Cohort of the Malmö Diet and Cancer study (MDC-CC). The top SNPs were then replicated in another non-overlapping cohort, the Malmö Preventive Project (MPP). The final step would be to genotype the best performing SNP in more than 24,000 people in the whole Malmö Diet and Cancer study (MDC) and relate it to known GH-associated variables such as height and BMI. In the second approach, which was a candidate gene approach, we analyzed SNPs in the growth hormone gene (*GH1)* [10] and the growth hormone receptor gene (*GHR)* [11] in the MDC-CC with subsequent replication in the MPP and MDC as previously described.

## Methods

### Cohorts

#### MDC-CC – Discovery cohort

The Malmö Diet and Cancer study (MDC) is a Swedish population-based, prospective study to which inhabitants in Malmö born between 1923 to 1945 (Males), or 1923 to 1950 (Females), were invited to participate. Twenty-eight thousand four hundred forty-nine accepted and attended a baseline examination between 1991 and 1996. A random 50% sample of the participants examined in MDC between 1991 and 94 (*n* = 12,445) were invited to also participate in a study on the epidemiology of carotid artery disease, the cardiovascular cohort of the MDC (MDC-CC). Six thousand one hundred three subjects accepted and underwent an even more detailed examination, which has been previously described [2, 12]. After overnight fasting, blood samples were drawn between 7.30 a.m. and 9.00 a.m. from 5540 individuals and stored immediately at −80 °C. GH was measured in the stored plasma samples with a high-sensitivity chemiluminescence sandwich immunoassay (SphingoTec GmbH, Borgsdorf, Germany) previously described [2]. The analytical assay sensitivity (mean relative light units of 20 determinations of GH free sample plus 2 S.D.; limit of detection, LOD) was 0.002 μg/L GH. The functional assay sensitivity (< 20% inter assay CV; limit of quantification, LOQ) was 0.01 μg/L. GH concentration above the LOQ (0.01 μg/L) were measured with an interassay precision of typically below 10% CV. The assay was calibrated using dilutions of GH (NIBSC code 98/574, National Institute for Biological Standards and Control, Herfordshire, UK). Individuals with a high-sensitive GH (hs-GH) value of 0 were censored in the analyses (*n* = 12). Genotyping of the MDC-CC was made using the Human Omni Express plus exome Bead Chip and iScan system (Illumina, San Diego, CA, USA) analyzing 850,000 common and rare SNPs. Five thousand four hundred fifty-one individuals were successfully genotyped, i.e. past our QC criteria (Additional files 1, 2 and 3). Values of hs-GH were available in 4134 of these (58% females; age 46 to 68 yrs).

#### MPP – Replication cohort

The Malmö Preventive Project (MPP) is a Swedish single-center, prospective, population-based study. A total of 33,346 men and women from the Malmö area with a homogeneous ethnic background, were recruited and screened for traditional risk factors of all-cause mortality and cardiovascular disease between 1974 and 1992 [13, 14]. Between 2002 and 2006, all survivors from the original MPP cohort were invited for a follow-up examination. Eighteen thousand two hundred forty participants (6682 women) accepted the invitation. The risk factors were re-assessed and after overnight fasting blood samples were drawn between 7.30 a.m. and 9.00 a.m. and immediately stored at −80 °C [15]. Levels of hs-GH were measured with the same methodology as in MDC-CC in a random sample (*n* = 5419) and successful in 5268 individuals. The only exclusion criterion before the random selection was participation (*n* = 9736) in MDC (and thus also MDC-CC) in order to generate non-overlapping cohorts (Additional file 1: Table S1). Genotyping in the subset was made with TaqMan (Applied Biosystems, Foster City, CA, USA) with primers and conditions according to the manufacturer’s recommendations. Genotyping of at least 1 SNP was successful, i.e. past our QC criteria, in 5413 individuals. The replication cohort consists of those in which both genotyping and measurement of hs-GH was successful (*n* = 5262; 28.9% females; age 56 to 85 yrs).

#### MDC – Anthropometric replication cohort

As previously mentioned in the MDC-CC-section, the Malmö Diet and Cancer study (MDC) is a prospective population-based cohort of 28,449 individuals examined 1991–96. Details about the examination can be found in previous publications [16]. Height, weight, and waist were measured with shoes and trousers off. Height was measured to the nearest cm and weight to the nearest 0.1 kg. Non-fasting blood samples were drawn and stored in −80 °C. Genotyping of the SNPs obtained from the results in MDC-CC and MPP was made using TaqMan (Applied Biosystems, Foster City, CA, USA) with primers and conditions according to the manufacturer’s recommendations. NB, a subset of participants (*n* = 5451) was already genotyped in MDC-CC with the Human OmniExpress Bead Chip as outlined in the previous section. Genotyping of at least 1 SNP was successful, i.e. past our QC criteria, in 28,181 individuals, 4134 of these were included in the GWAS in MDC-CC and were thus excluded from the replication sample in MDC, which then consisted of 24,047 individuals.

### Genetic analyses

**Fig. 1:**
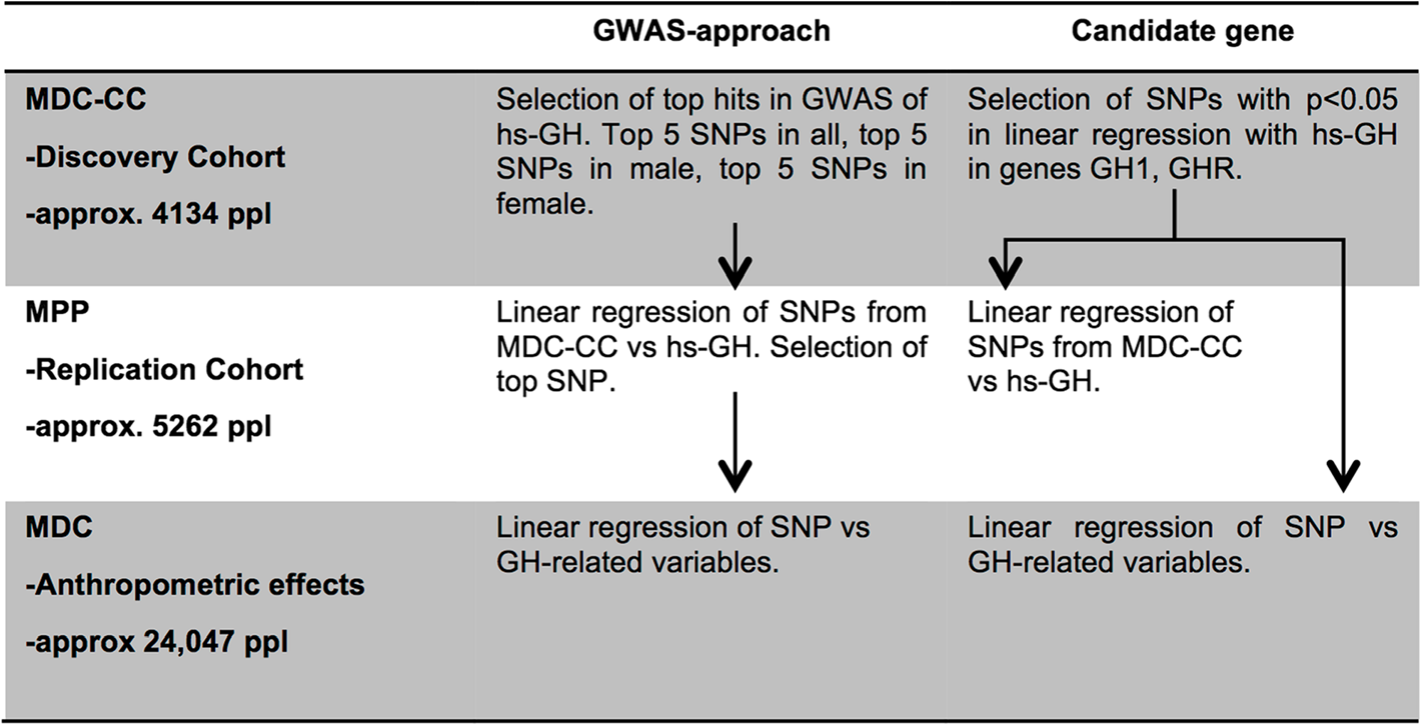
Overview of planned methodology in the GWAS and candidate gene approaches

An overview of the planned methodology can be found in Fig. 1. Where applicable the effect of the alleles where assumed to be additive in the linear regression models.

### GWAS-approach

In the discovery analyses in **MDC-CC**, we applied the SNP inclusion criterion of minor allele frequency (MAF) ≥ 0.01, which was fulfilled in 659,208 of the genotyped SNPs. Multivariate linear regression models with log-transformed hs-GH as the dependent variable were executed for each of the 659,208 SNPs and adjusted for gender and age using PLINK software [17]. The 5 SNPs with the lowest *p*-value in each associated locus were selected for replication in MPP. In addition, in a gender-stratified analysis the top 5 SNPs in males and females were also selected for replication based on the same criteria. We confirmed that the Hardy-Weinberg assumption was met in all the selected SNPs.

In **MPP** each of the selected SNPs from the previous step were included in linear regression models with the standardized value of the natural logarithm of hs-GH as the dependent variable and the SNP, sex, and age as independent variables. The SNP with the overall lowest p-value, based on both combined and gender-stratified analysis, was selected for further study on GH-related traits in the whole MDC-cohort.

In the **MDC** study, blood samples at baseline were not drawn after an overnight fast and thus measurement of fasting hs-GH was not possible. Instead we used several variables that are known to be associated with fasting hs-GH [2, 18] such as height, body mass index (BMI), and waist. Separate linear regression models were performed with each of the variables as dependent and the SNP, sex, and age as independents. Models were also performed gender-stratified.

The impact of the top SNP on cardiovascular disease was evaluated through a search in the publically available Cardiogram database (http://www.cardiogramplusc4d.org/). Cardiogram is a collaborative effort of multiple large-scale genetic studies with the aim to identify risk loci for coronary artery disease and myocardial infarction [19].

### Candidate gene

In **MDC-CC**, age- and gender-adjusted linear regression models with hs-GH as dependent variable was performed as previously described. We analyzed the SNPs annotated to the genes GH1 and GHR that had a MAF ≥ 0.01 and selected the SNPs with a *p*-value < 0.05 for replication in MPP. When SNPs were in LD (r^2^ > 0,75 in spearman correlation) with each other, the SNP with the lowest p-value or with fewest missing genotypes in the genotyping were chosen. We confirmed that the Hardy-Weinberg assumption was met in all the selected SNPs.

The selected SNPs were then genotyped in **MPP **and analyzed vs the fasting value of hs-GH in age- and gender-adjusted linear regression models followed by tests of the top-SNP from MDC-CC against anthropometric traits in the whole **MDC** study.

Analyses in MPP and MDC performed in SPSS (version 22.0.0, SPSS Inc., Chicago, Ill). A 2-sided *P* value of less than 0.05 was considered nominally significant. P for difference in Table 1 was calculated with student’s t-test (mean values), chi-square test (proportions) or Mann-Whitney test (GH). In some instances a bonferroni-corrected significance level was calculated by dividing 0.05 with the number of independent tests performed.

**Table 1:** Baseline characteristics of eligible participants in the different cohorts

	MDC-CC - Discovery	MPP - Replication	MDC - Anthropometry
Number of participants	4134	5262	24,047
Female (%)	2405 (58.2)	1522 (28.9)***	14,588 (60.7)**
Age, mean (SD), years	57.7 (6.0)	69.3 (6.3)***	58.1 (7.9)**
Body Mass Index, mean (SD), kg/m2	25.8 (3.9)	27.2 (4.2)***	25.8 (4.1)
Waist, mean (SD), cm	83.8 (12.9)	95.8 (12.2)***	84.5 (13.1)**
Waist-hip-ratio (SD)	0.85 (0.09)	0.93 (0.09)***	0.85 (0.09)
Bodyfat percentage, mean (SD), %	27.6 (7.1)	N/A	26.8 (7.0)***
Height, mean (SD), cm	169 (9)	171 (9)***	169 (9)
Diabetes Mellitus (%)	367 (8.9)	615 (11.7)***	1043 (4.3)***
Smoking (%)	1081 (26.1)	1028 (19.5)***	6338 (26.4)
Lipid-lowering therapy (%)	89 (2.2)	1060 (20.1)***	762 (3.2)***
Growth Hormone - males, median (IQR), μg/L	0.11 (0.06–0.33)	0.40 (0.11–1.27)***	N/A
Growth Hormone - females, median (IQR), μg/L	1.21 (0.38–3.12)	0.49 (0.14–1.62)***	N/A

*p < 0.05 for difference compared with MDC-CC. **p < 0.01 for difference compared with MDC-CC. ***p < 0.001 for difference compared with MDC-CC

## Results

Baseline characteristics of the three different cohorts are shown in Table 1. There were more males in MPP compared with the other cohorts. The participants in MPP were also older, had a larger waist and were more likely to be on lipid lowering therapy. The differences in hs-GH between males and females are less pronounced in MPP when compared with MDC-CC. As stated in the methods section, 53% of the MPP-cohort participated in MDC and was excluded before the random selection for the hs-GH and genetic analyses, baseline characteristics of these are available in Additional file 1: Table S1.

### GWAS-approach

In the GWAS in MDC-CC 659,208 SNPs were available for analysis after QC control. There was no indication of population stratification and the median genomic inflation factor (lambda) was 1.0 (for Q-Q plot see Additional file 2: Figure S1). The SNP with the strongest association with hs-GH in the gender-combined analyses in MDC-CC was rs7920826 with a *p*-value of 4,24 × 10^−6^ (Table 2). The top 5 SNPs from all, top 5 from male and top 5 from female (Additional file 1: Table S2) were selected for replication in MPP. The SNP rs7208736, which will be of special interest in this article, was associated with fasting hs-GH in all (Beta [SD-increase of log-GH per 1 minor allele], −0.145; 95%CI, −0.207 to −0.082; *p* = 5.15 × 10^−6^), in males (Beta, −0.095; 95%CI, −0.187 to −0.003; *p* = 0.04), and in females (Beta, −0.180; 95%CI, −0.264 to −0.096; *p* = 2.67 × 10^−5^). No SNP reached the genome-wide significance level of 5 × 10^−8^. A Manhattan plot is included in the supplementary material (Additional file 3: Figure S2).

**Table 2:** Fifteen selected SNPs in relation to hs-GH in GWAS-approach in MDC-CC

SNP	Chr	Basepair^a^	Major allele	Minor allele	MAF	Gene^b^	β	95%CI	P
rs7920826	10	122,808,053	T	G	0.16	–	−0.19	−0.27 to −0.11	4.2^a^10^-6
rs9816337	3	28,936,109	G	T	0.46	–	−0.14	−0.20 to −0.08	4.3^a^10^-6
rs7208736	17	1,807,705	G	A	0.36	–	−0.14	−0.21 to −0.08	5.2^a^10^-6
rs6552287	4	179,194,375	T	C	0.42	–	−0.14	−0.20 to −0.08	5.2^a^10^-6
rs10513091	5	11,423,525	T	C	0.15	CTNND2	0.19	0.11 to 0.27	6.5^a^10^-6
rs11644234	16	53,036,844	C	T	0.11	–	−0.21	−0.30 to −0.11	1.1^a^10^-5
rs3803071	12	94,698,880	T	C	0.45	PLXNC1	−0.13	−0.19 to −0.07	1.3^a^10^-5
rs10472071	5	58,031,027	A	G	0.09	RAB3C	0.22	0.11 to 0.32	5.3^a^10^-5
rs17094404	8	13,370,143	T	C	0.03	DLC1	0.26	0.09 to 0.42	0.003
rs7987689	13	43,928,135	C	T	0.34	ENOX1	−0.09	−0.15 to −0.03	0.005
rs4839595	3	143,163,169	G	A	0.45	SLC9A9	0.08	0.02 to 0.14	0.008
rs6767160	3	71,741,068	T	C	0.06	EIF4E3	0.15	0.02 to 0.28	0.018
rs687543	5	81,220,187	C	T	0.10	–	−0.12	−0.21 to −0.02	0.019
rs2361028	1	119,410,215	G	T	0.45	–	0.04	−0.02 to 0.10	0.14
rs16961034	16	59,419,461	T	C	0.01	–	0.30	0.03 to 0.57	0.030

Minor allele modeled as effect allele.The β coefficients are expressed as the increment of standardized values of the natural logarithm of hs-GH per 1 *minor* allele. Adjusted for age and sex

*Abbreviations*: *chr* Chromosome number, *MAF* Minor allele frequency, *Gene* Annotated gene. No available for analysis in: all: 4123–4134

^a^basepair positions refer to GRCh37

^b^Gene annotation

The 15 SNPs from MDC-CC were genotyped in the replication cohort MPP (Additional file 1: Table S3). The minor allele of rs7208736 exhibited a significant negative association in the MPP replication cohort (direction same as in MDC-CC) with fasting hs-GH in all (Beta [SD-increase of log-GH per 1 minor allele], −0.056; 95%CI, −0.095 to −0.017; *p* = 0.005) and in males (Beta, −0.061; 95%CI, −0.107 to −0.015; *p* = 0.009), but not in females (Beta, −0.043; 95%CI, −0.116 to 0.030; *p* = 0.25) and was selected for further investigation in MDC. No other of the 15 GWAS-derived SNPs replicated in the MPP (Additional file 1: Tables S3 and S4).

Thus, the SNP rs7208736 was subsequently genotyped in the whole MDC study in order to analyze the SNP vs anthropometric variables (Table 3). In linear regression models the minor allele, which was associated with lower hs-GH, was significantly associated with lower BMI (*p* = 0.026) and lower waist (*p* = 0.021) in males, but not in all or in females.

**Table 3:** Linear regression models of the effect of the two selected SNPs on GH-related variables in MDC

		rs7208736 (GWAS)			rs13153388 (Candidate gene)		
	Variable	Beta	95%CI	P	Beta	95%CI	P
All	Height	−0.002	−0.015 to 0.011	0.78	−0.019	−0.032 to −0.006	**0.003**
	BMI	−0.005	−0.024 to 0.014	0.59	0.009	−0.010 to 0.027	0.36
	Waist	−0.004	−0.019 to 0.012	0.64	0.010	−0.005 to 0.025	0.18
Male	Height	−0.006	−0.036 to 0.024	0.70	−0.017	−0.046 to 0.012	0.26
	BMI	−0.035	−0.065 to −0.004	**0.026**	0.010	−0.020 to 0.039	0.51
	Waist	−0.036	−0.066 to −0.005	**0.021**	0.008	−0.021 to 0.038	0.58
Female	Height	0.000	−0.024 to 0.024	0.99	−0.034	−0.057 to −0.011	**0.003**
	BMI	0.009	−0.015 to 0.033	0.46	0.008	−0.015 to 0.032	0.48
	Waist	0.013	−0.011 to 0.037	0.28	0.015	−0.008 to 0.039	0.19

Minor allele modeled as effect allele.One linear regression model is executed per variable with the variable in question as independent. Adjusted for sex and age. Beta coefficients are expressed as the increment of standardized values of the variable (BMI, waist, height) per 1 minor allele. Individuals available for analysis in rs7208736 ranging between 23,126–23,140 in all; 9103–9109 in males and 14,023–14,031 in females. Individuals available for analysis in rs13153388 ranging between 22,997–23,011 in all; 9024–9031 in males and 13,973–13,980 in females. *p*-values < 0.05 in bold

### Coronary artery disease

Since high levels of hs-GH have been associated with cardiovascular disease we investigated the relation between coronary artery disease and rs7208736 in Cardiogram, which is a meta-analysis of 22 GWAS studies with participants of European descent [19]. The allele associated with higher hs-GH (***major*** allele, G) in our data was associated with an increase in the odds ratio of CAD with 1.05 (95%CI 1.01–1.09; *P* = 0.01) per 1 allele in 10,327 cases and 30,178 controls.

### Candidate gene approach

We also performed a candidate gene approach in our search for genetic determinants of the fasting level of hs-GH. SNPs in the genes GH1 and GHR were genotyped and then related against the fasting level of hs-GH. In our assay there were 2 SNPs in the gene GH1, however both of these had a MAF < 0.01 which was our limit for further analysis. In the gene GHR there were 49 SNPs, but in 17 of these the MAF was below 0.01. Thus we could analyze 32 SNPs vs hs-GH. Among the whole cohort, 8 SNPs were nominally significant (*p* < 0.05). After stratification by sex, 9 SNPs were nominally significant in males and none were significant in females. Six of the nominally significant SNPs in all and male overlapped. After LD-analysis, four of the SNPs with independent signals in all were selected for replication in MPP (Table 4). The top SNP was rs13153388, where the minor allele was associated with increased hs-GH in the male (Beta [SD-increase of log-GH per 1 minor allele], 0.116; 95%CI, 0.029 to 0.204;*p* = 0.009) and gender-combined (Beta, 0.088; 95%CI, 0.029 to 0.147; *p* = 0.003) analyses, but not among females (Beta, 0.066; 95%CI, −0.014 to 0.145; *p* = 0.11). Three of the four selected SNPs were nominally significant in males as well (Additional file 1: Table S5). Of note is that the SNP rs6873545, which is a marker for GHRd3 [20], was not associated with the fasting levels of hs-GH in neither combined (*p* = 0.95) nor gender-stratified analyses (males, *p* = 0.80; females, *p* = 0.85).

**Table 4:** Nominally significant SNPs in GHR-gene in MDC-CC

SNP	Basepair^a^	Major allele	Minor allele	MAF	Beta	95%CI	P
rs13188386	42,473,555	G	A	0.26	0.085	0.018 to 0.151	0.01
rs13153388	42,507,333	T	G	0.44	0.088	0.029 to 0.147	0.003
rs4365846	42,547,255	A	G	0.24	0.087	0.018 to 0.156	0.01
rs6883523	42,564,748	G	T	0.18	0.079	0.003 to 0.155	0.04

Minor allele modeled as effect allele.The β coefficients are expressed as the increment of standardized values of the natural logarithm of hs-GH per 1 minor allele. Adjusted for age and sex

*Abbreviations*: *MAF* Minor allele frequency.Number of individuals available for analysis: 4126–4131

^a^Basepair number for polymorphism (GRCh37). All the SNPs are located in the GHR-gene on chromosome 5

The four selected SNPs were then genotyped in MPP in a replication effort. However none of the candidate gene SNPs were significant in linear regression models vs hs-GH. Including both combined and gender-stratified analyses, the lowest *p*-value was 0.18 in the gender-combined analysis of rs13188386.

**Fig. 2:**
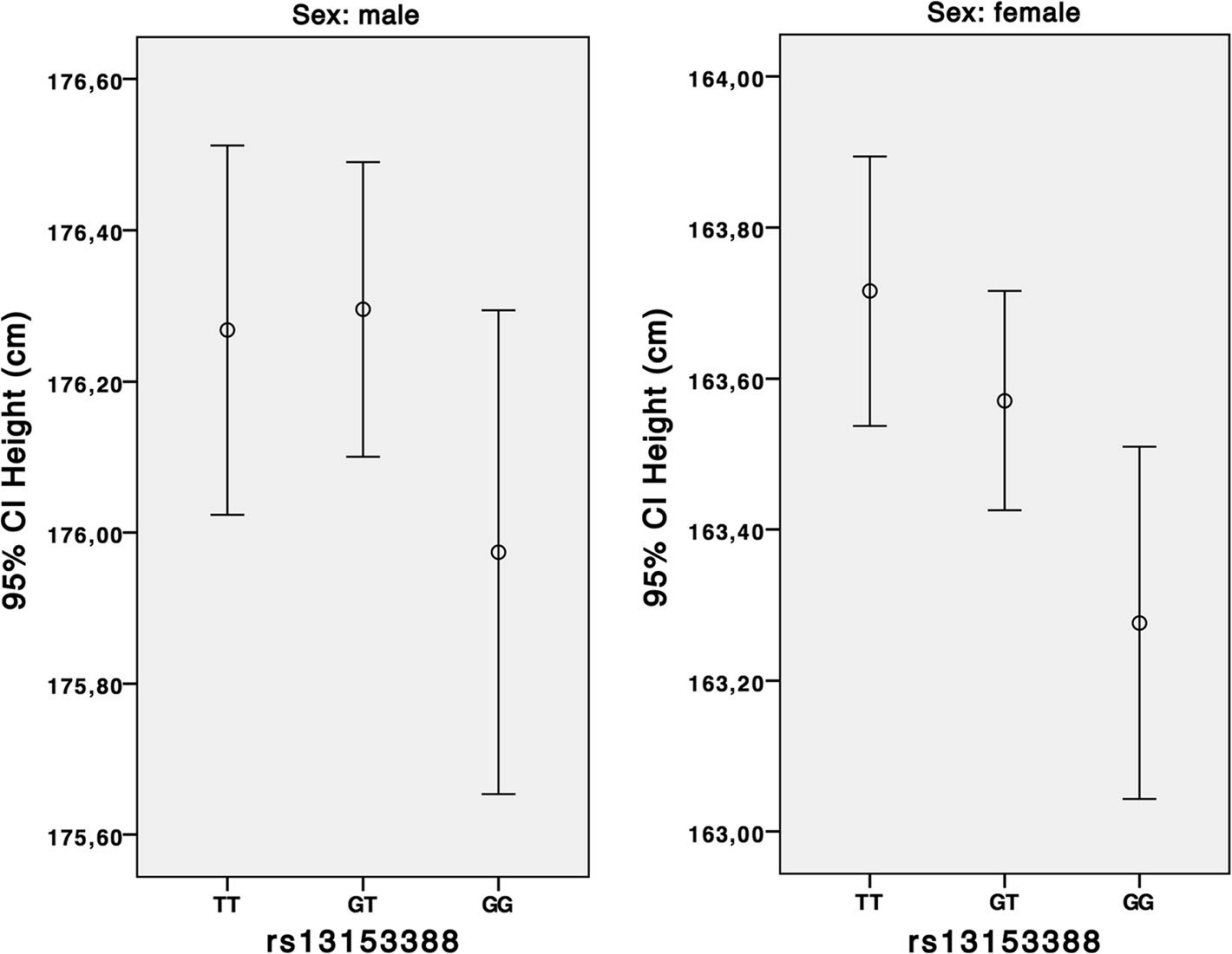
Height of the participants in the MDC study stratified on number of minor allele (G) copies in the SNP rs13153388, which is located in the GHR-gene. Number of individuals in analysis, males: TT, 2970; GT, 4432; GG, 1647. Females: TT, 4667; GT, 6807; GG, 2529

The top SNP from MDC-CC, rs13153388, was genotyped in MDC to relate it to anthropometric variables in multivariate linear regression models adjusted for sex and age (Table 3). The GH-increasing minor allele of rs13153388 was significantly associated with lower height in all (*p* = 0.003), and females (*p* = 0.003), but not in males (*p* = 0.26). Females were approximately 0,2 cm shorter per 1 minor allele (Fig. 2). It is noteworthy that the association with height is negative in a SNP that is associated with a higher level of hs-GH, which will be further discussed in the next section.

## Discussion

This is the first genome-wide analysis on the fasting level of GH that has been conducted, and the largest genetic study on the fasting level of GH in all categories. A GWAS-approach with a discovery phase in 4134 individuals and subsequent replication in a non-overlapping cohort of 5262 individuals identified one SNP on chromosome 17 in which the minor allele was associated with a reduced fasting level of hs-GH. A candidate gene approach identified SNPs in the GHR associated with the fasting level of hs-GH, which in a later phase showed strong associations to height. The main clinical implication of our findings is that genetic signals, such as those identified in the current study, are necessary to be able to assess whether the previously reported associations between high circulating GH levels in the fasted state and risk of cardiovascular morbidity and mortality are causal or not in Mendelian Randomization studies. Also, the novel locus associated with the fasting level of GH represents a completely new biological mechanism behind control of plasma GH level that deserves further study.

The minor allele of rs7208736 was associated with a significant reduction of the fasting level of hs-GH both in the discovery and replication cohort. The results were stronger in females in MDC-CC, but stronger in males in MPP. It should be noted that there were approximately half as many women in MPP as in MDC-CC, which may explain part of the reduced performance among females in MPP. Although the minor allele was associated with a reduction of hs-GH, it was also associated with a lower BMI and waist in the male part of MDC, which may seem counter intuitive since these variables are inversely related to the fasting level of hs-GH [2, 18]. An augmenting modification of the effect of hs-GH could explain the results, since this would lead to larger effects on target tissues and reduced secretion due to enhanced feedback signaling, i.e. the opposite of GH-insensitivity syndrome [21, 22]. It should however be noted that the associations between rs7208736 and BMI and waist are rather weak for such a big cohort, which underlines the need for replication of our results, especially concerning the metabolic effects of this SNP.

Rs7208736 is located in a non-coding sequence of the short arm of chromosome 17 (17p13.3). The GH-gene cluster is located on the same chromosome, but on the other arm, so any direct interaction with this gene is unlikely. However, numerous genes, which the SNP might affect through enhancers or regulatory motifs, are located nearby. Inactivation of the region around rs7208736 has been linked to several malignancies, such as lung, breast, liver, colon, kidney, and brain, with the genes HIC1 (Hypermethylated in cancer 1) and OVCA2 (Ovarian cancer-associated gene 2) being potential tumor suppressors [23, 24]. HIC1 is located approximately 150 kbp downstream of rs7208736, and could be the link between rs7208736 and GH. Among other effects, HIC1 represses transcription of SIRT1 (Sirtuin 1) [23, 25]. SIRT1 is a deacetylase that is activated by caloric restriction and in vivo in rats have been shown to negatively regulate GH-dependent IGF-1 production in the liver by deacetylation of STAT5 [26]. STAT5 is a downstream target of GHR-signaling via JAK2-activation [11]. Thus higher levels of HIC1 would inhibit transcription of SIRT1, which could augment the effect of GHR-signaling and would explain the picture with the minor allele in rs7208736 reducing levels of hs-GH but at the same time also being associated with lower BMI and waist. However this is very speculative, but nevertheless provides a plausible mechanism with support in the literature. The associations are intriguing and further studies will be needed to replicate this finding and determine the underlying pathways.

When investigated in Cardiogram the *major* allele of rs7208736, which is associated with a higher level of hs-GH, was associated with an OR of 1.05 per major allele for incident coronary artery disease. This is in line with previously published results of higher levels of hs-GH being associated to cardiovascular disease [2, 3]. However one could argue that it does not fit with the suggested theory in the previous section, i.e. the *minor* allele being associated with augmented sensitivity in the GHR-signaling. It should be noted that Cardiogram included more individuals than MDC, which may suggest that our proposed mechanism in with the minor allele of rs7208736 augmenting GH-signaling may be erroneous. Alternately you could interpret the results as reduced GH-signalling being associated with CVD, and our previous findings being explained as a compensatory mechanism [2]. This emphasizes the need for replication and further studies on the subject.

In the candidate gene approach the minor allele in rs13153388 was associated with a higher hs-GH and lower height. Height is as expected positively associated with hs-GH [18]. If the SNP is associated with a reduced function of the GHR, the consequence could be a mild form of GH-insensitivity syndrome [21, 22], with a reduced effect of hs-GH, i.e. short stature, but, due to reduced feedback signaling, still high levels of hs-GH in plasma. The effect of the SNP on adult height is small, but evident (Fig. 2).

Unfortunately, the results in the GHR-gene did not replicate in MPP. This may be attributable to differences in the composition of the cohorts. When comparing the baseline characteristics between MPP and MDC-CC, the population in MPP is older, consists of more males and a larger proportion are on medications that potentially could affect GH-levels. The fasting levels of hs-GH display a more pronounced gender difference in MDC-CC than in MPP. Notably the reduced difference in MPP is attributable to both an increase in males and a decrease in females. The gender difference in the fasting levels has previously been described to decline with age [27]. Among females, menopausal status and hormone replacement therapy might also affect the decline in hs-GH, since estrogen is positively associated with hs-GH [28]. It may be that these confounding effects have diluted the differences in GH-levels and made associations harder to capture. However there is indication of more long-term effects of GH in the anthropometric analyses with the strong connection to height, which is heritable to about 80% [29], implying that the SNP might affect the levels of GH earlier in life.

The study has some limitations. There was a fair amount of non-responders to the invitations, which may have biased selection of a more healthy population. Also, in MPP, roughly half the participants were excluded before the random selection, since they participated in the MDC (Additional file 1: Table S1). This was necessary to obtain independent cohorts, but also may have biased MPP-cohort, since willingness to participate in studies (i.e. likelihood to participate in both studies) infers a positive health selection, which could reduce the healthiest part of the cohort in the current study. The pulsatile secretion of hs-GH make measurements more difficult, but as previously discussed we have tried to eliminate this issue by drawing blood in the morning hours when the levels are more stable [2, 6–8]. It is inevitable that samples are taken from some individuals during a peak, but given the large number of participants it is still possible to analyze hs-GH on a group level. A single fasting value of hs-GH is not a standard clinical test. The correlation between fasting GH and 24-h GH secretion is strong, but with large variability [9]. However, on a population basis, a single fasting value of hs-GH strongly and independently predicts cardiovascular disease and death as we have previously shown [2], which calls for further research on the subject.

As discussed previously there were some differences in the characteristics between the MDC-CC and MPP. However this should be an advantage to evaluate if the results are applicable to a broader population. A concern could be that the individuals in MPP were old and that the function of their GH-IGF-1-axis may have been reduced.

In the GWAS-approach we did not reach genome-wide significance in our analysis, which means that the results have to be interpreted cautiously. However we managed to replicate our findings in a separate cohort, which increases the credibility of the results. This underlines the need of further replication of our results in independent cohorts. Since the genetic variant is not confirmed it is difficult to assess any causality of GH in cardiovascular disease through a Mendelian randomization.

In the candidate gene approach 32 SNPs were included in the initial analysis, a bonferroni-corrected *p*-value would be *P* = 0.002 (0.05/32), which the SNP rs13153388 misses by a fine margin. This emphasizes the need for further replication.

Both cohorts (MDC and MPP) are from the same geographic area with the same genetic background and allele frequencies of the studied SNPs were similar in all cohorts. This makes the results difficult to extrapolate to other ethnic groups. The results will need to be replicated in larger studies, as well as in populations with another genetic background to attain a larger generalizability.

## Conclusions

In conclusion, we discovered one SNP (rs7208736) on chromosome 17 where presence of the minor allele is associated with a reduced fasting level of hs-GH. In the GHR we found one variant associated with a higher fasting level of hs-GH and lower height. Our findings need further replication in independent cohorts. If true they point at novel biological mechanisms underlying control of fasting plasma GH level and will facilitate future Mendelian Randomization efforts of investigating whether the previously described relationship between GH and cardiovascular morbidity and mortality is causal or not.

## Additional files

Additional file 1: Hallengren_supp.docx. QC-protocol, supplementary file 1–5. (DOCX 34 kb)

Additional file 2: Figure S1 Quantile–quantile plot of the genome-wide association analysis for fasting levels of hs-GH in MDC-CC [median genomic inflation factor (lambda) = 1.0] (PNG 63 kb)

Additional file 3: Figure S2. Manhattan plot of GWAS in MDC-CC relating SNPs with the fasting level of hs-GH. *P*-values for the association between SNPs and the natural logarithm of the fasting level of hs-GH. Blue line represents a significance level of 5 × 10^−5^. None of the SNPs reached the significance level 5 × 10^−8^. (PNG 582 kb)

## Abbreviations

CV: Coefficient of variation
CVD: Cardiovascular disease
GH: Growth Hormone
GHR: Growth Hormone Receptor
GWAS: Genome-wide association study
hs-GH: High-sensitivity Growth Hormone
LD: Linkage disequilibrium
MAF: Minor allele frequency
MDC: Malmö Diet and Cancer study
MDC-CC: Malmö Diet and Cancer study, cardiovascular cohort
MPP: Malmö Preventive Project
QC: Quality control
SNP: Single Nucleotide Polymorphism
