# Supplementary material for: Genetic determinants of growth hormone and GH-related phenotypes
Source: BMC Genomics. 2017 Oct 24;18:822. doi: 10.1186/s12864-017-4219-z (PMC5655832; doi:10.1186/s12864-017-4219-z)
Supplement: Supplementary file 1 — Hallengren_supp.docx. QC-protocol, supplementary file 1–5. (DOCX 34 kb) [file 12864_2017_4219_MOESM1_ESM.docx]

## SUPPLEMENTARY MATERIAL

## Quality control criteria in GWAS in MDC-CC

Individual level QC was performed by excluding individuals with

- call rate <95%
- inbreeding coefficient 0.2 or higher
- disconcordant sex in self-report vs genetically determined sex
- a second degree relatedness or higher within the sample, based on identity by descent sharing calculations
- individuals that were population outliers based on inspection of the first two principal component plots.

In marker level QC, we excluded variants with:

- call rate <95%
- variants on sex chromosomes
- mitochondrial DNA
- variants showing an extreme deviation from Hardy-Weinberg equilibrium (P < 1 × 10^–6^).

**Table S1:** Baseline characteristics from MPP of individuals participating in both MDC and MPP (excluded in MPP-analyses) vs individuals only participating in MPP (before random selection for the study cohort).

| **Variable** | **Excluded (in MDC&MPP)** | **Only in MPP*** |
| --- | --- | --- |
| Number | 9736 | 8504 |
| Females | 44.7% | 27.4% |
| Age (males), years | 69.9 (4.8) | 66.9 (6.9) |
| Age (females), years | 69.8 (4.7) | 68.9 (5.2) |
| Smoking (males) | 14% | 21% |
| Smoking (females) | 13% | 20% |
| BMI (males), mean (SD), kg/m2 | 27.2 (3.8) | 27.4 (4.7) |
| BMI (females), mean (SD), kg/m2 | 26.9 (4.6) | 27.1 (4.8) |
| Height (males), mean (SD), cm | 175 (7.0) | 176 (6.8) |
| Height (females), mean (SD), cm | 162 (5.9) | 162 (6.9) |
| Waist (males), mean (SD), cm | 98.6 (10.4) | 99.3 (10.6) |
| Waist (females), mean (SD), cm | 86.9 (11.8) | 88.1 (12.0) |

*****5,419 of these were randomly selected for measurement of fasting hs-GH and genotyping.

**Table S2**: Gender–stratified GWAS in MDC-CC. Top 5 SNPs in each gender in bold.

|  | Male | | | Female | | |
| --- | --- | --- | --- | --- | --- | --- |
|  | Beta | 95%CI | P | Beta | 95%CI | P |
| rs7920826 | -0.20 | -0.32 to -0.08 | 8.4*10^-4 | -0.17 | -0.28 to -0.06 | 0.002 |
| rs9816337 | -0.10 | -0.19 to -0.02 | 0.017 | -0.16 | -0.24 to -0.08 | 7.6*10^-5 |
| rs7208736 | -0.09 | -0.19 to 0.00 | 0.043 | -0.18 | -0.26 to -0.10 | 2.7*10^-5 |
| rs6552287 | -0.16 | -0.25 to -0.08 | 2.5*10^-4 | -0.12 | -0.20 to -0.04 | 0.005 |
| rs10513091 | 0.14 | 0.02 to 0.27 | 0.020 | 0.22 | 0.11 to 0.33 | 9.2*10^-5 |
| rs11644234 | -0.01 | -0.15 to 0.13 | 0.91 | **-0.34** | **-0.46 to -0.22** | **5.2*10^-8** |
| rs3803071 | -0.03 | -0.12 to 0.05 | 0.46 | **-0.20** | **-0.28 to -0.12** | **4.8*10^-7** |
| rs10472071 | 0.04 | -0.11 to 0.19 | 0.61 | **0.36** | **0.21 to 0.50** | **1.8*10^-6** |
| rs17094404 | **0.59** | **0.35 to 0.83** | **1.5*10^-6** | -0.03 | -0.26 to 0.20 | 0.81 |
| rs7987689 | **-0.21** | **-0.30 to -0.12** | **8.0*10^-6** | 0.00 | -0.09 to 0.08 | 0.91 |
| rs4839595 | -0.05 | -0.14 to 0.03 | 0.23 | **0.17** | **0.09 to 0.25** | **1.5*10^-5** |
| rs6767160 | **0.49** | **0.29 to 0.68** | **1.5*10^-6** | -0.05 | -0.21 to 0.11 | 0.56 |
| rs687543 | **-0.32** | **-0.46 to -0.18** | **9.0*10^-6** | 0.04 | -0.09 to 0.18 | 0.53 |
| rs2361028 | **0.21** | **0.12 to 0.30** | **2.8*10^-6** | -0.08 | -0.16 to 0.00 | 0.056 |
| rs16961034 | -0.37 | -0.76 to 0.01 | 0.058 | **0.85** | **0.47 to 1.22** | **1.0*10^-5** |

The β coefficients are expressed as the increment of standardized values of the natural logarithm of hs-GH per 1 minor allele. Adjusted for age.

Individuals available for analysis ranging between 1,725-1,729 in males and 2,397-2,405 in females.

**Table S3:** Linear regression of GWAS-SNPs vs fasting levels of hs-GH in MPP.

| SNP | n | Beta | 95%CI | P |
| --- | --- | --- | --- | --- |
| rs7208736 | 5194 | -0.056 | -0.095 to -0.017 | 0.005 |
| rs3803071 | 5205 | 0.037 | 0.000 to 0.075 | 0.052 |
| rs11644234 | 5208 | 0.054 | -0.005 to 0.113 | 0.075 |
| rs9816337 | 5196 | 0.031 | -0.007 to 0.069 | 0.11 |
| rs6552287 | 5186 | 0.024 | -0.014 to 0.063 | 0.21 |
| rs687543 | 5157 | -0.038 | -0.102 to 0.027 | 0.25 |
| rs4839595 | 5128 | .019 | -0.019 to 0.057 | 0.32 |
| rs6767160 | 5210 | -0.029 | -0.105 to 0.048 | 0.46 |
| rs10472071 | 5207 | 0.020 | -0.045 to 0.086 | 0.54 |
| rs17094404 | 5218 | 0.028 | -0.072 to 0.128 | 0.58 |
| rs7920826 | 5156 | 0.014 | -0.038 to 0.065 | 0.60 |
| rs10513091 | 5213 | 0.012 | -0.041 to 0.065 | 0.65 |
| rs7987689 | 5207 | 0.008 | -0.032 to 0.048 | 0.69 |
| rs16961034 | 5201 | -0.025 | -0.201 to 0.151 | 0.78 |
| rs2361028 | 5182 | 0.001 | -0.037 to 0.039 | 0.96 |

The β coefficients are expressed as the increment of standardized values of the natural logarithm of hs-GH per 1 minor allele. Adjusted for age and sex.

**Table S4**: Gender–stratified replication of GWAS-SNPs in MPP.

|  | Male | | | Female | | |
| --- | --- | --- | --- | --- | --- | --- |
|  | Beta | 95%CI | P | Beta | 95%CI | P |
| rs10472071 | 0.016 | -0.062 to 0.095 | 0.68 | 0.029 | -0.091 to 0.150 | 0.63 |
| rs10513091 | 0.026 | -0.036 to 0.088 | 0.41 | 0.026 | -0.036 to 0.088 | 0.41 |
| rs11644234 | 0.035 | -0.034 to 0.105 | 0.32 | 0.102 | -0.011 to 0.215 | 0.077 |
| rs16961034 | -0.011 | -0.206 to 0.185 | 0.92 | -0.087 | -0.496 to 0.323 | 0.68 |
| rs17094404 | 0.024 | -0.095 to 0.143 | 0.69 | 0.037 | -0.149 to 0.224 | 0.69 |
| rs2361028 | 0.018 | -0.027 to 0.063 | 0.43 | -0.040 | -0.111 to 0.030 | 0.26 |
| rs3803071 | 0.045 | 0.001 to 0.090 | **0.047** | 0.018 | -0.053 to 0.089 | 0.62 |
| rs4839595 | 0.00 | -0.045 to 0.045 | 0.99 | 0.069 | -0.003 to 0.141 | 0.06 |
| rs6552287 | 0.019 | -0.027 to 0.064 | 0.42 | 0.038 | -0.033 to 0.109 | 0.30 |
| rs6767160 | -0.059 | -0.149 to 0.032 | 0.20 | 0.044 | -0.098 to 0.186 | 0.54 |
| rs687543 | -0.012 | -0.086 to 0.063 | 0.76 | -0.118 | -0.248 to 0.013 | 0.077 |
| rs7208736 | -0.061 | -0.107 to -0.015 | **0.009** | -0.043 | -0.116 to 0.030 | 0.25 |
| rs7920826 | 0.031 | -0.030 to 0.092 | 0.31 | -0.029 | -0.124 to 0.067 | 0.55 |
| rs7987689 | 0.007 | -0.041 to 0.054 | 0.78 | 0.012 | -0.063 to 0.087 | 0.76 |
| rs9816337 | 0.027 | -0.018 to 0.072 | 0.23 | 0.040 | -0.032 to 0.112 | 0.27 |

The β coefficients are expressed as the increment of standardized values of the natural logarithm of hs-GH per 1 minor allele. Adjusted for age.

Individuals available for analysis ranging between 3639-3709 in males and 1489-1511 in females.

**Table S5:** Gender stratified results for SNPs in the GHR-gene selected for replication.

|  | **SNP** | **N*** | **Beta**** | **95%CI** | **p** |
| --- | --- | --- | --- | --- | --- |
| Male | rs13188386 | 1727 | 0.109 | 0.010 to 0.207 | .03 |
|  | rs13153388 | 1727 | 0.116 | 0.029 to 0.204 | 0.009 |
|  | rs4365846 | 1727 | 0.105 | 0.002 to 0.207 | .04 |
|  | rs6883523 | 1726 | 0.055 | -0.058 to 0.167 | .34 |
| Female | rs13188386 | 2404 | 0.066 | -0.024 to 0.157 | .15 |
|  | rs13153388 | 2403 | 0.066 | -0.014 to 0.145 | .11 |
|  | rs4365846 | 2399 | 0.075 | -0.017 to 0.168 | .11 |
|  | rs6883523 | 2403 | 0.097 | -0.005 to 0.199 | .06 |

*Number available for analysis.

**The β coefficients are expressed as the increment of standardized values of the natural logarithm of hs-GH per 1 minor allele. Adjusted for age.
