# Supplementary figures and images for: Genetic determinants of growth hormone and GH-related phenotypes
Source: BMC Genomics. 2017 Oct 24;18:822. doi: 10.1186/s12864-017-4219-z (PMC5655832; doi:10.1186/s12864-017-4219-z)

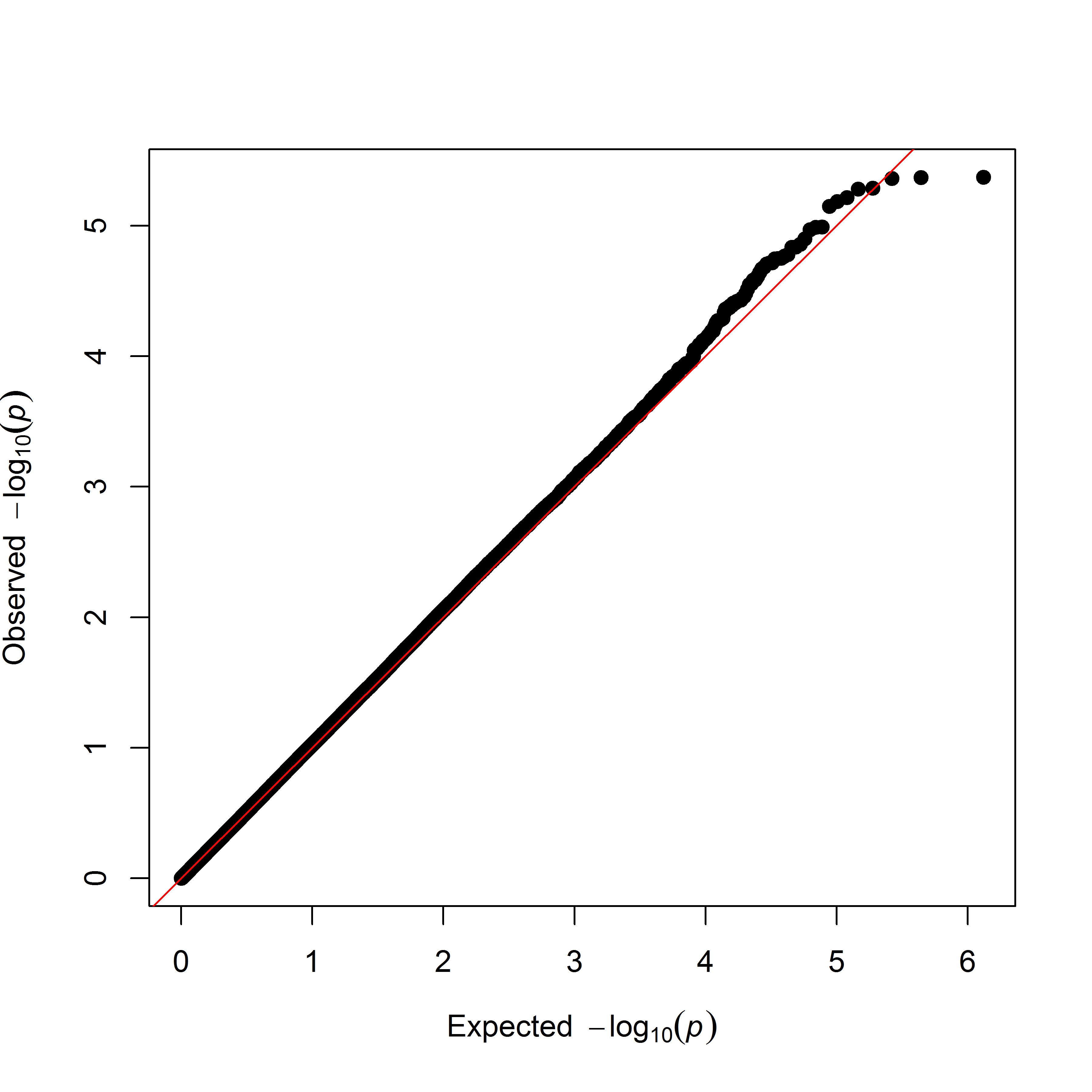

Supplement: Supplementary file 2 — Quantile–quantile plot of the genome-wide association analysis for fasting levels of hs-GH in MDC-CC [median genomic inflation factor (lambda) = 1.0] (PNG 63 kb) [file 12864_2017_4219_MOESM2_ESM.png]

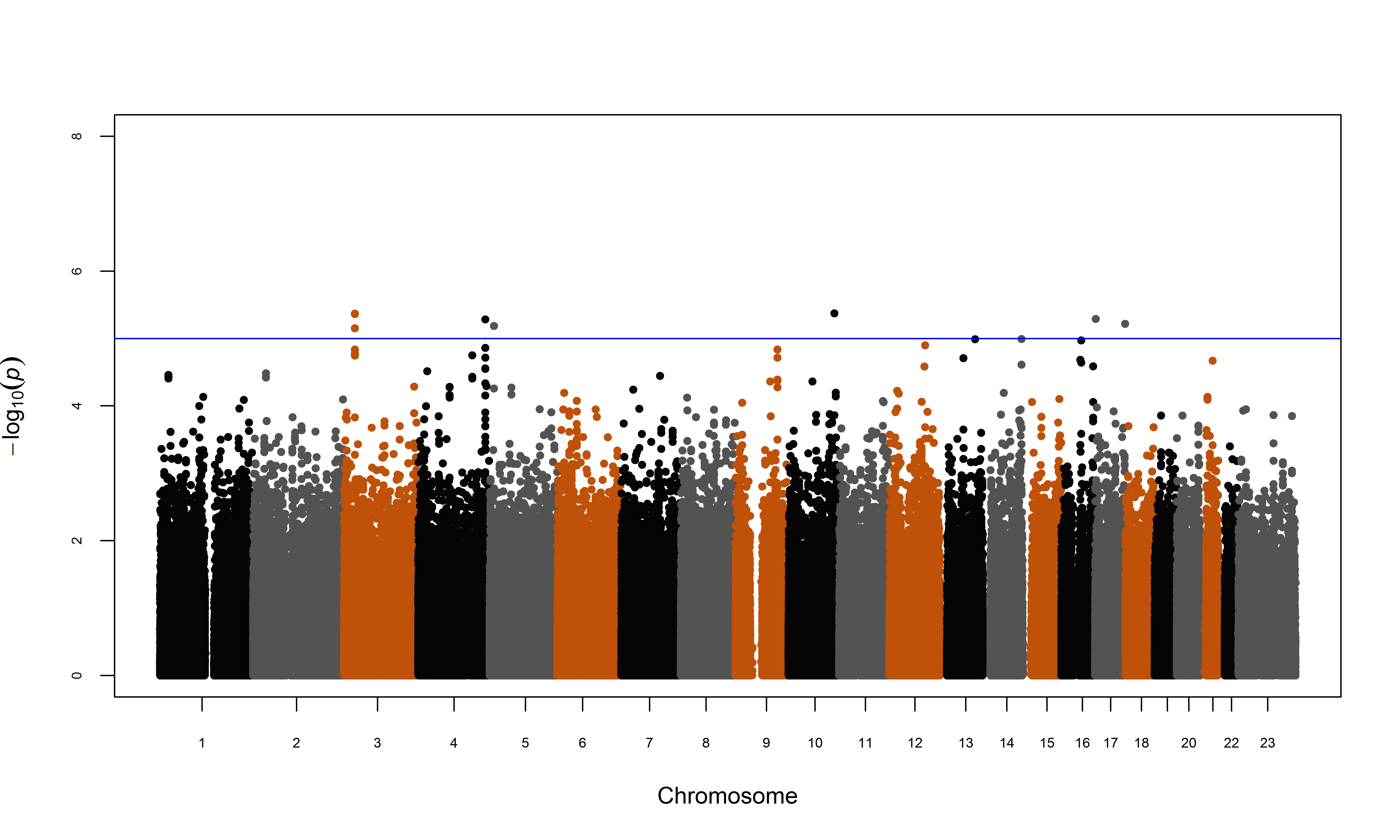

Supplement: Supplementary file 3 — Manhattan plot of GWAS in MDC-CC relating SNPs with the fasting level of hs-GH. P-values for the association between SNPs and the natural logarithm of the fasting level of hs-GH. Blue line represents a significance level of 5 × 10^−5^. None of the SNPs reached the significance level 5 × 10^−8^. (PNG 582 kb) [file 12864_2017_4219_MOESM3_ESM.png]
